# Supplementary material for: Viral Diversity in Samples of Freshwater Gastropods Benedictia baicalensis (Caenogastropoda: Benedictiidae) Revealed by Total RNA-Sequencing
Source: Int J Mol Sci. 2023 Nov 30;24(23):17022. doi: 10.3390/ijms242317022 (PMC10707223; doi:10.3390/ijms242317022)
Supplement: Supplementary file 1 [file ijms-24-17022-s001.zip › Supplementary Figure S1.pdf]

Supplementary Figure

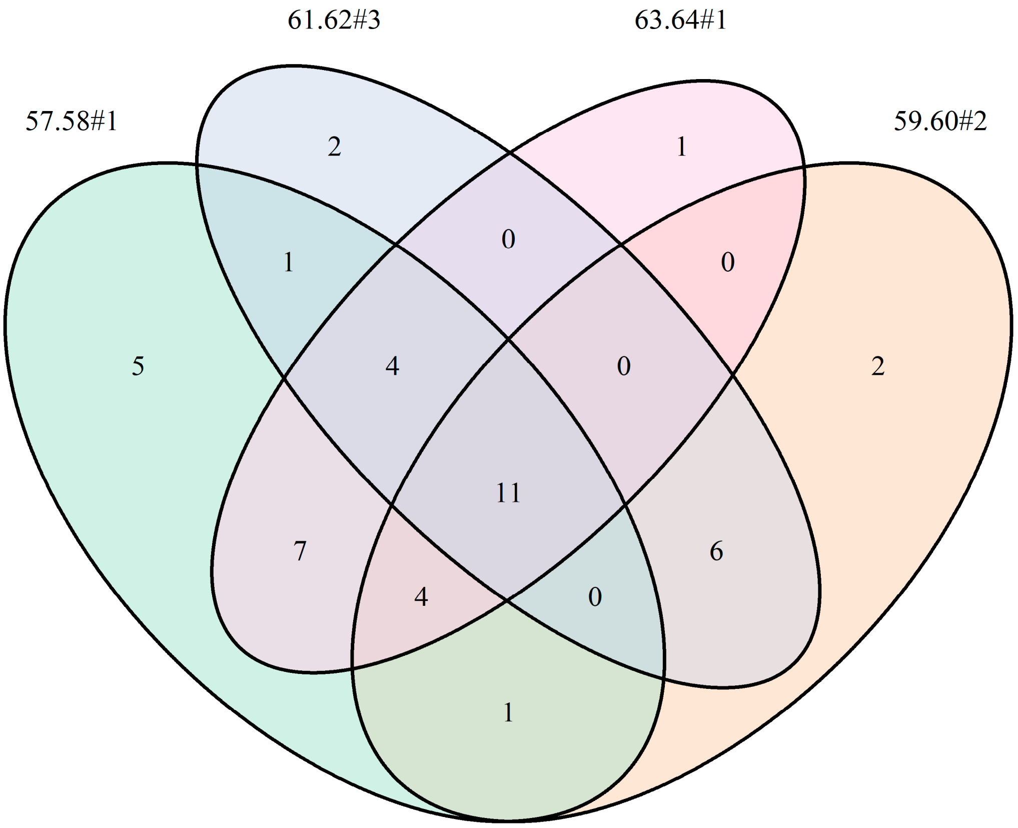

Figure S1. A Venn diagram showing the similarity of samples based on the number of common virotypes (constructed using the vegan package for R).
